# Quantifying mitral regurgitation in MVP: the added value of three-dimensional continuity equation

**DOI:** 10.1093/ehjimp/qyag009

**Published:** 2026-01-15

**Authors:** Abdalla Eltayeb A Abdelkader, Amro Alsaid, Ralph Matar, Prajakta Phatak, Janaki Rami Reddy Manne, Zuyue Wang

**Affiliations:** Division of Cardiovascular Medicine, University of Florida, 1600 SW Archer Rd, Gainesville, FL 32610, USA; Baylor Scott & White, The Heart Hospital, Plano, TX, USA; Baylor Scott & White, The Heart Hospital, Plano, TX, USA; Baylor Scott & White, The Heart Hospital, Plano, TX, USA; Baylor Scott & White, The Heart Hospital, Plano, TX, USA; Baylor Scott & White, The Heart Hospital, Plano, TX, USA

**Keywords:** mitral valve prolapse, continuity equation, volumetric assessment, three-dimensional echocardiography, cardiac magnetic resonance

## Abstract

**Aims:**

Quantifying mitral regurgitation (MR) in patients with mitral valve prolapse (MVP) is particularly challenging due to the complex mitral valve anatomy, presence of multiple eccentric jets, and non-holosystolic regurgitation. The continuity equation (CE) offers a potentially more accurate method for assessing MR regurgitant volume in this population.

**Methods and results:**

We evaluated patients with MVP and at least moderate MR. Regurgitant volumes (RVol) were quantified using the three-dimensional continuity equation (3D-CE) with direct area measurements and compared with RVol derived from the conventional diameter-based continuity equation (D-CE) and cardiovascular magnetic resonance (CMR). Among 72 patients (mean age 59.7 years, 65% female), bileaflet MVP was present in 86% and MAD in 96%. Multiple MR jets (32%) limited PISA accuracy (*r* = 0.40 with CMR). Compared with 3D-CE, D-CE overestimated RVol by 19 mL, though correlation remained strong (*r* = 0.74). In 21 paired studies, 3D-CE and CMR showed excellent agreement (*r* = 0.94, bias +2.1 mL). Severity grading showed strong concordance, with most discrepancies within one category.

**Conclusion:**

In patients with MVP, incorporating 3D annular measurements into 3D-CE improves agreement with CMR-based volumetric assessment and mitigates overestimation associated with D-CE and PISA-based methods. This approach may be particularly valuable in patients with moderate or moderate-severe MR, in whom conventional echocardiographic parameters are frequently discordant, and supports the role of integrative multimodality imaging in refining MR severity assessment and guiding clinical management. Larger prospective studies are warranted to confirm these findings.

## Graphical Abstract

**Graphical Abstract:**
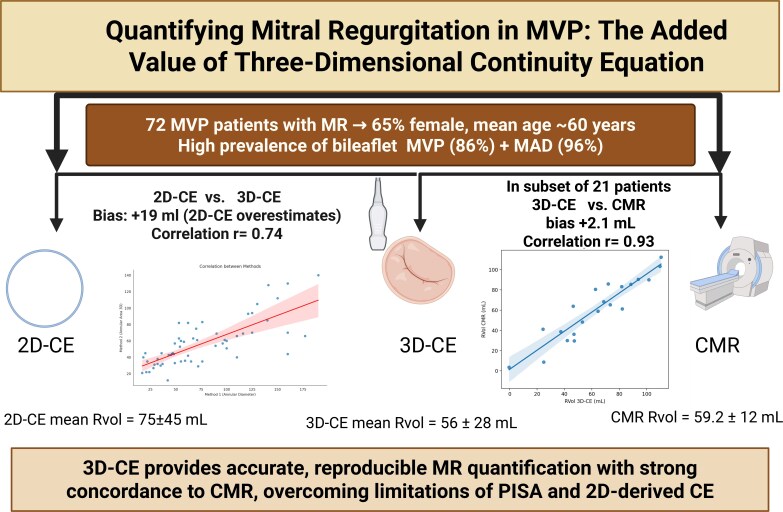

## Introduction

Mitral valve prolapse (MVP), affecting approximately 2% of the general population, is the most common cause of primary mitral regurgitation (MR).^1^ In patients with MVP, MR severity is frequently misclassified or inconsistently graded, contributing to delays in diagnosis and referral for timely intervention. Such delays may result in irreversible left ventricular (LV) remodelling and are associated with increased morbidity and mortality.^2,3^

Moderate to severe MR affects approximately 1.5 million individuals in the United States.^4^ Accurate quantification of MR in MVP remains challenging owing to eccentric or multiple regurgitant jets, which limit the reliability of conventional echocardiographic methods such as the proximal isovelocity surface area (PISA) and vena contracta. In addition, chronic MR alters left atrial (LA) compliance and chamber volumes, thereby reducing the utility of qualitative parameters, such as pulmonary vein flow patterns, for severity assessment.^5^

The continuity equation offers a quantitative approach to MR assessment based on principles of flow conservation.^6^ However, its application is limited by the geometric assumption of circular mitral annular (MA) and left ventricular outflow tract (LVOT) areas when derived from a single diameter. This assumption is particularly problematic in the MVP setting, where the MA is often non-circular, with intercommissural dimensions exceeding anteroposterior diameters, potentially leading to systematic errors in regurgitant volume estimation.^7^

This study aims to improve MR quantification in patients with MVP by incorporating three-dimensional (3D) assessment of the MA and LVOT areas into the continuity equation (3D-CE). In accordance with guideline-recommended integrative assessment of MR severity, we sought to determine whether 3D-CE provides greater agreement with cardiac magnetic resonance (CMR)–derived volumetric assessment than conventional echocardiographic methods, particularly in patients with borderline severe and moderate MR. More refined MR classification in this subgroup may have important implications for clinical management, including appropriate timing of intervention and intensity of longitudinal surveillance. Accordingly, we compared 3D-CE with the diameter-derived continuity equation (D-CE) and PISA-based quantification, using CMR volumetric assessment as the reference standard.

## Methods

We retrospectively enrolled all consecutive patients with MVP with at least moderate MR who underwent transthoracic echocardiography (TTE) and transoesophageal echocardiography (TEE) from 2023 to 2024 at Baylor Scott & White—The Heart Hospital in Plano, Texas. Clinical and imaging data were collected for analysis. In a subset of patients who underwent CMR imaging within 10 days of echocardiography and in whom no interim clinical or haemodynamic changes were documented, regurgitant volume (RVol) derived from 3D-CE was compared with RVol obtained by CMR-based volumetric assessment. Patients were excluded if there was a suboptimal image, if CE-based MR quantification could not be performed due to concomitant valvular disease (including aortic regurgitation, aortic stenosis, or mitral stenosis), or if essential data were unavailable, such as pulsed-wave Doppler (PW) measurements at the MA or LVOT, or 3D echocardiographic datasets. Additional exclusion criteria included the presence of non-MVP cardiac conditions such as intracardiac shunts, congenital heart disease, hypertrophic cardiomyopathy, or high-output states like pregnancy. The final cohort consisted of patients with isolated MVP. As this was a retrospective study, the institutional review board waived the requirement for written informed consent, and the study protocol was approved.

### Echocardiography

A comprehensive transthoracic (TTE) and transoesophageal echocardiographic (TEE) assessment was performed according to the American Society of Echocardiography (ASE) guidelines by a Level 3 echocardiographer blinded to prior findings.^8,9^ MR severity was evaluated using the ASE-recommended integrative approach. Standard parameters included RVol and effective regurgitant orifice area (EROA) calculated by the PISA method, vena contracta width, LA volume, LV dimensions, and pulmonary vein systolic flow. A focused TTE was conducted on the same day as the TEE, using parasternal long- and short-axis and apical two-, three-, and four-chamber views. PISA measurements were obtained from multiple TEE views with a Nyquist limit of 32 cm/s. Continuous-wave Doppler was used to assess peak MR velocity and velocity-time integral (VTI). Pulmonary vein flow was assessed via pulsed-wave Doppler in both the right and left pulmonary veins, when feasible. Measurements essential for the CE, including LVOT diameter and PW velocities at both the LVOT and mitral annulus levels, were acquired to estimate RVol by subtracting forward stroke volume (mitral annular area × mitral VTI) from total LV stroke volume (LVOT area × LVOT VTI).^6^ MA diameters were obtained from the mid-oesophageal transoesophageal echocardiographic window. The anteroposterior annular diameter was measured in the mid-oesophageal long-axis view (0°), and the orthogonal diameter was measured in the mid-oesophageal commissural view (approximately 60°–90°). Measurements were performed at both end-diastole and end-systole to account for the dynamic nature of the mitral annulus throughout the cardiac cycle. For diameter-derived continuity equation (D-CE) calculations, the average of the two annular diameters was used to mitigate potential under- or overestimation of annular area in the setting of a noncircular and dynamically changing mitral annulus. LVOT diameter was measured in mid-systole using the inner-edge to inner-edge methodology in the mid-oesophageal long-axis view. For 3D-CE analysis, measurements of MA and LVOT were obtained offline from the 3D TEE datasets using TomTec 4D MV Assessment software (TomTec Imaging Systems, Unterschleissheim, Germany). Datasets were aligned in orthogonal planes, and semi-automated annular tracing was performed across multiple views with manual refinement as needed. RVol was then calculated using the 3D annular area (3D-CE) and compared with conventional diameter-derived area (D-CE) estimates to assess the impact of geometric assumptions on MR quantification. All measurements were independently reviewed, and intra- and interobserver variability was evaluated in a subset to confirm reproducibility. Additionally, mitral leaflet length, LV dimensions, and the presence and extent of MAD were evaluated. MAD was defined as a distinct separation between the mitral annulus and the basal LV wall, visible in parasternal long-axis TTE or the corresponding three-chamber TEE view.^10^ MAD length was measured in end-systole from the annular hinge point to the crest of the LV wall.^11^

### Cardiac magnetic resonance (CMR)

CMR was performed on a 1.5T Achieva scanner (Philips Medical Systems) using software platforms 5.6.1 or 5.6.1.3. Multiplanar, multisequence CMR was performed using gradient-echo cine imaging to assess cardiac morphology and function, supplemented by phase-velocity (flow) sequences to quantify blood flow velocities. The imaging included steady-state free precession (SSFP) cine sequences acquired in standard short-axis slices spanning from the mitral annulus to the LV apex at 1 cm intervals, with a slice thickness of 6 mm and no interslice gap. Additional long-axis views (2-, 3-, and 4-chamber) were obtained to evaluate mitral annular plane systolic excursion and global LV function. Phase-contrast (PC) imaging was performed to measure aortic flow at the sinotubular junction, with velocity encoding settings optimized between 150 and 200 cm/sec to minimize aliasing. Temporal and spatial resolution parameters were adjusted according to the protocol to achieve high-fidelity flow data. RVol was calculated as the difference between total LV stroke volume (derived from manual contouring of end-diastolic and end-systolic volumes on cine images) and forward stroke volume across the aortic valve from PC imaging, following Society of Cardiovascular Magnetic Resonance guidelines.^12^ This adherence to guidelines ensures the validity and reliability of the results. Regurgitant fraction (RF) was computed as RVol divided by total stroke volume. All image post-processing was conducted on a dedicated workstation using Cvi42 software (Circle Cardiovascular Imaging Inc., Release Version 5.113.5). Gadolinium-based contrast (Gadavist) was administered intravenously, and delayed-enhancement imaging was performed according to the standard protocol to assess myocardial viability. Additionally, parametric mapping was acquired and analysed to characterize tissue properties. In patients with MVP, prolapsed volume was quantified by tracing the displaced leaflet area during systole on 3D SSFP cine images across orthogonal planes, and integrating this volume into the RVol calculation to account for leaflet redundancy contributing to regurgitation.^13,14^

### Statistical analysis

All measurements from echocardiography and CMR were collected, paired, and cleaned by removing missing values to ensure completeness across both modalities. Continuous variables were reported as mean ± standard deviation or as median with 25th and 75th percentiles, as appropriate. Categorical data were presented as absolute numbers or percentages. To compare measurements between echocardiography and/or CMR, paired Student's *t*-tests were performed, with statistical significance set at *P* < 0.05. For continuous data, the Student's *t*-test and Mann–Whitney *U* test were used to compare two groups of unpaired data with Gaussian and non-Gaussian distributions, respectively. Agreement between modalities was assessed using Bland–Altman plots, which illustrated the mean difference (bias) and the 95% limits of agreement (±1.96 standard deviations from the mean difference), with the x-axis representing the mean of paired measurements and the y-axis displaying the difference between them. To evaluate the strength and direction of the linear relationship between echocardiography and/or CMR measurements, Pearson's correlation coefficient (*r*) was computed along with corresponding *P*-values to assess statistical significance. Correlation plots, including the line of identity, were generated to visualize these relationships. Categorical variables were analysed using the chi-square test. All statistical analyses were conducted using Python (version 3.11).

## Result

The baseline characteristics of the 72 patients are summarized in *Tables 1* and *2*. The mean age was 59.7 ± 14.5 years; 49 patients (65.3%) were female, and bileaflet MVP was present in 62 patients (86%). MAD was identified in 69 (95.8%) of the cohort, with a mean distance of 8.0 ± 3.0 mm by echocardiography and 8.52 ± 3.00 mm by CMR.

**Table 1:** Baseline patient characteristics (*N* = 72)

**Demographics**	
Age, year	59.7 ± 14.48
Sex, Female	49 (65.3%)
Height, cm	171 ± 10
Weight, kg	71 ± 14.5
BSA, m^2^	1.8 ± 0.23
SBP, mmHg	120 ± 20
SBP, mmHg	73 ± 12
HR, bpm	71 ± 15
**Echocardiography**	
LA diameter (AP), cm	3.9 ± 0.83
LA volume indexed to BSA, cm/m^2^	45.7 ± 22.3
LVIDD, cm	5.1 ± 0.8
LVIDS, cm	3.4 ± 0.83
IVSd, cm	1.07 ± 0.75
PWD, cm	1.02 ± 0.19
EDV, ml	115.7 ± 34.48
EDV indexed to BSA, mL/m²	63.72 ± 18.12
ESV, mL	54.31 ± 30.13
ESV indexed to BSA, mL/m²	29.44 ± 14.87
EF, %	58 ± 9.2
GLS, %	−16.8 ± 4.85
LVOT, cm	2.2 ± 0.27
LVOT area by 3D planimetry, cm2	4.1 ± 0.7
LVOT VTI, cm/sec	18.4 ± 4.2
MV VTI, cm/sec	14.4 ± 3.1
Presence of multiple MR jets	23 (32%)
Presence of bileaflet MVP	62(86%)
Presence of flail MV leaflet	7 (10%)
MV annulus by 3D planimetry	9.5 ± 2.5
VC, mm	0.56 ± 0.15
MR RVol of CE-3D, ml	56 ± 28
MR RVol of CE-D, ml	75 ± 45
MR RVol by PISA, ml	53 ± 39
Mitral annulus diameter, cm	3.8 ± 0.68
**Cardiac magnetic resonance (CMR)**	
LA diameter, cm	4.0 ± 0.6
LA volume indexed to BSA cm/m2	54.3 ± 27
IVSd cm	0.94 ± 0.28
PWDd cm	0.78 ± 0.26
LVEDV, ml	176 ± 53
LVEDV indexed to BSA, mL/m²	95 ± 22.5
LVESV, ml	72 ± 27
LVESV indexed to BSA, mL/m²	38 ± 13
LVEF, %	60 ± 6.7
RVEDV, ml	149 ± 44
RVEDV indexed to BSA, mL/m²	84 ± 26
RVESV, ml	72 ± 29
RVESV indexed to BSA, mL/m²	41 ± 18
RVEF,%	53 ± 7.6
MR RVol, ml	59.2 ± 12
Post contrast T1 relation time, ms	1054 ± 57
T2	49 ± 9.8
ECV,	29.6 ± 3.1
Presence of fibrosis in inferolateral wall, %	30 (47.6%)
Presence of fibrosis in papillary muscles, %	12 (19.0%)
Presence of diffuse fibrosis, %	5 (7.9%)

Abbreviations: BSA, body surface area; SBP, systolic blood pressure; HR, heart rate; LA, left atrium; LVIDD, left ventricular internal diameter in diastole; LVIDS, left ventricular internal diameter in systole; IVSd, interventricular septal thickness in diastole; PWDd, posterior wall thickness in diastole; EDV, end-diastolic volume; ESV, end-systolic volume; EF, ejection fraction; GLS, global longitudinal strain; LVOT, left ventricular outflow tract; MV, mitral valve; TDI, tissue Doppler imaging; AML, anterior mitral leaflet; PML, posterior mitral leaflet; MVP, mitral valve prolapse; RVol, regurgitant volume; VC, vena contracta; MAD, mitral annular disjunction; PASP, pulmonary artery systolic pressure; TAPSE, tricuspid annular plane systolic excursion; ECV, extracellular volume; 3D-CE: three-dimensional (3D) assessment of the mitral annular area into the continuity equation (3D-CE); D-CE: conventional diameter-derived area (D-CE).

**Table 2:** Comparison between echocardiography and CMR

Variable	Echocardiography	MRI	*P*-value
PWTD, cm	1.10 ± 0.89	0.92 ± 0.94	0.334
IVSD, cm	1.01 ± 0.19	0.94 ± 0.26	**0.011**
LA (A-P) diameter, cm	3.72 ± 0.72	4.13 ± 0.69	0.108
LA/BSA, mL/m²	42.83 ± 18.78	52.88 ± 25.25	0.209
EDV/BSA, mL/m²	65.3 ± 19.1	93.7 ± 21.4	**0.001**
ESV/BSA, mL/m²	27.84 ± 9.85	36.70 ± 12.80	**0.001**
EF, %	58.70 ± 8.34	61.02 ± 6.55	**0.033**

Refer to *Table 1* for the abbreviations.

Multiple MR jets were observed in 23 patients (32%), which made MR quantification using the PISA method challenging and resulted in a modest correlation with CMR (*r* = 0.40).

**Figure 1:**
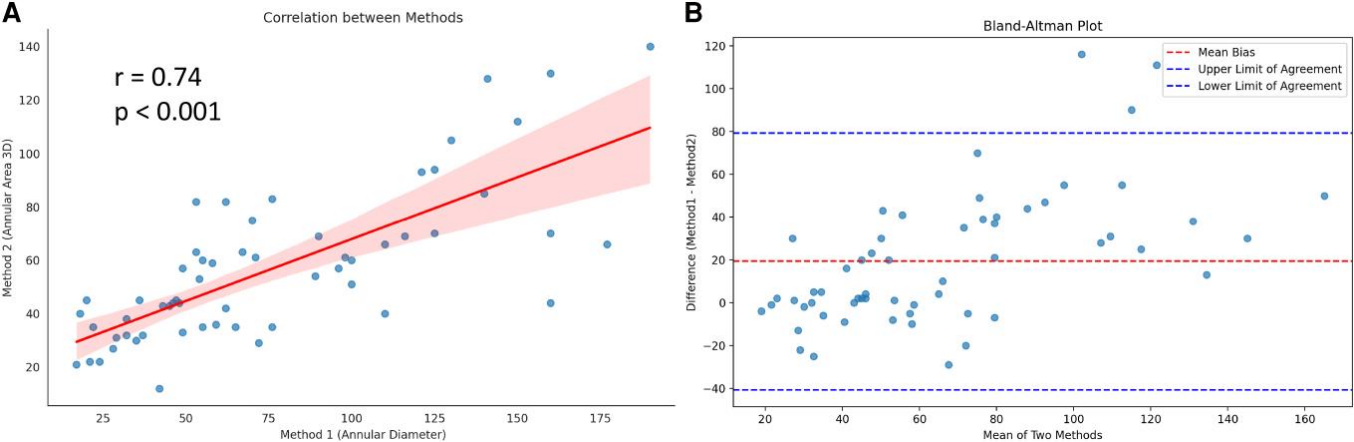
Comparison of regurgitant volumes (RVol) measured by three-dimensional continuity equation (3D-CE) and diameter-derived continuity equation (D-CE). **(***A***) Scatter plot:** Correlation between RVol measured by 3D-CE (x-axis) and D-CE (y-axis). A strong positive correlation is observed (Spearman’s ρ = 0.744, *P* < 0.001). Variability increases at higher RVol values, reflecting differences between methods. **(***B***) Bland–Altman plot:** Agreement between 3D-CE and D-CE for RVol. The y-axis represents the difference (3D-CE − D-CE), and the x-axis represents the mean RVol of the two methods. The solid line indicates the mean bias (−19.28 mL), and the dashed lines represent the 95% limits of agreement (−79.27 to +40.71 mL). Differences are more pronounced at higher regurgitant volumes.

Comparison of regurgitant volume between 3D-CE and D-CE revealed significant differences (*P* < 0.001), with D-CE systematically overestimating regurgitant volume by a mean bias of 19.3 mL. Despite a strong correlation (*r* = 0.74, *P* < 0.001), agreement was moderate to good (ICC = 0.67, 95% CI 0.50–0.79). Severity classification of RVol showed moderate agreement (Cohen's κ = 0.52), with perfect agreement in 71% of cases and most disagreements limited to adjacent categories (*Figure 1*). Reproducibility for 3D annular area measurement was good, with an ICC of 0.82.

**Figure 2:**
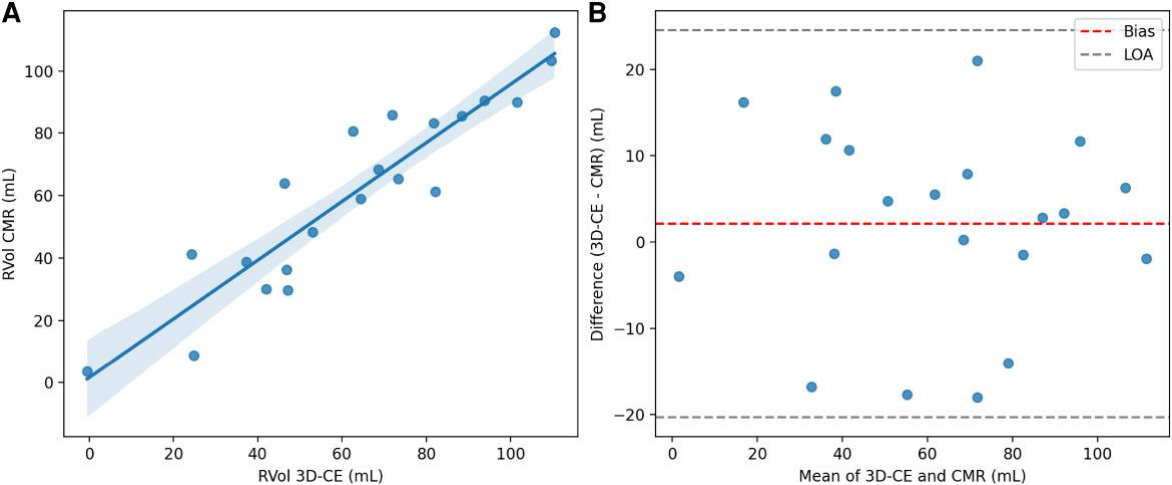
Comparison of regurgitant volume (RVol) measured by three-dimensional continuity equation (3D-CE) and cardiac magnetic resonance (CMR) in a subset of 21 patients. **(***A***) Scatter plot:** RVol measured by 3D-CE (x-axis) vs. CMR-derived RVol (y-axis) demonstrates a strong linear correlation (Pearson r ≈ 0.94, *P* < 0.001). Axes are labelled as ‘Mitral Regurgitant Volume by 3D-CE (mL)’ and ‘Mitral Regurgitant Volume by CMR (mL).’ **(***B***) Bland–Altman plot:** The difference between 3D-CE and CMR RVol (3D-CE − CMR) is plotted against the mean RVol of the two methods. Bland–Altman analysis showed a small positive bias of 2.1 mL, indicating slight overestimation by 3D-CE relative to CMR. The 95% limits of agreement ranged from −19.0 to +18.7 mL, reflecting good concordance across the measurement range.

**Figure 3:**
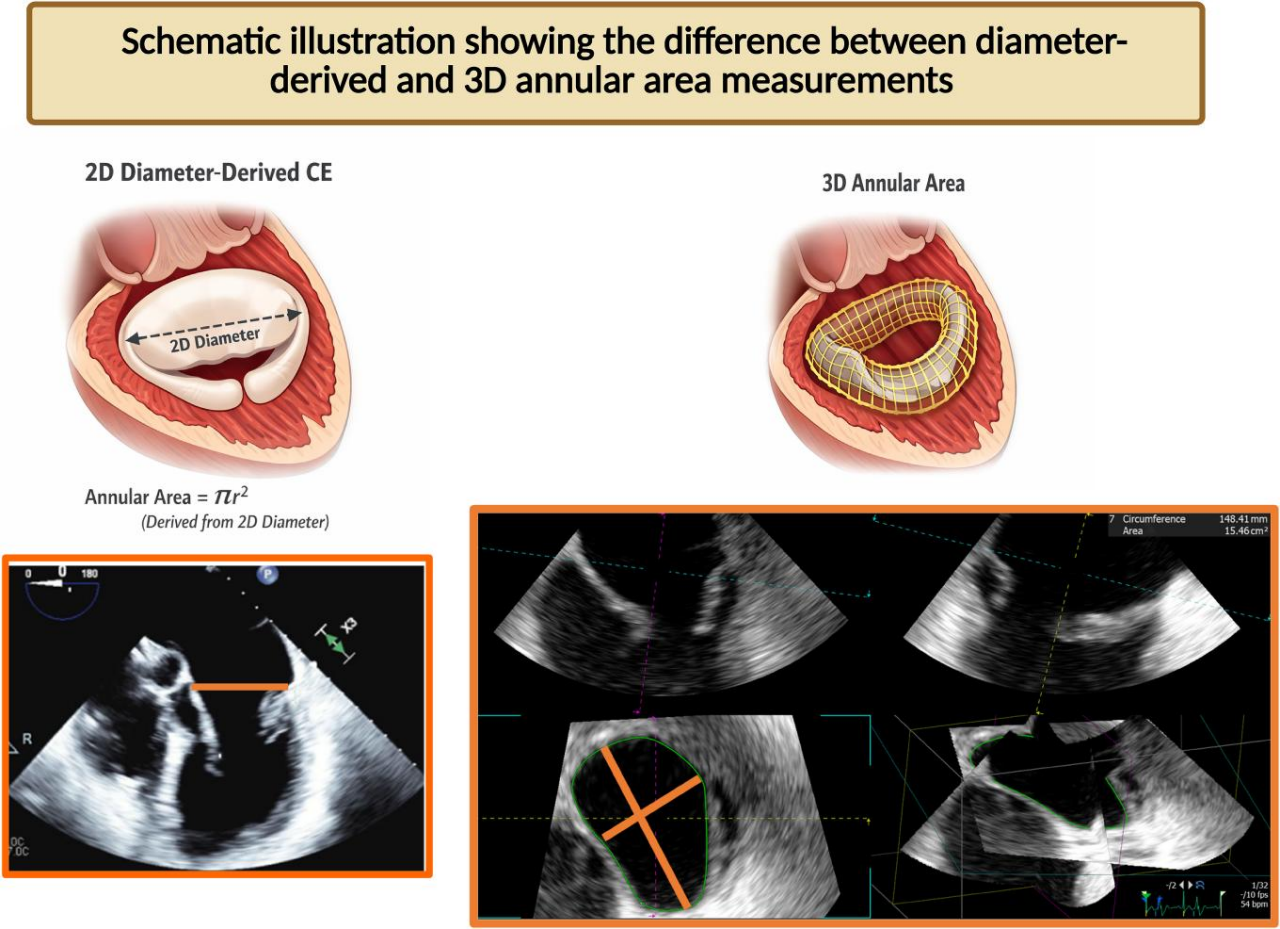
Representative case showing perfect agreement in mitral regurgitation quantification between 3D continuity equation and cardiac MRI.

Among the 21 patients who underwent echocardiography and CMR within 10 days, there were no interim clinical changes or interventions, and blood pressure and heart rate remained stable between studies with no significant differences observed (all *P* > 0.05). RVol showed good agreement between 3D-CE and CMR, with mean values of 56 ± 28 mL and 59.2 ± 12 mL, respectively. Pearson correlation demonstrated an excellent linear relationship (*r* = 0.93, *P* < 0.001) with an intraclass correlation coefficient using a two-way mixed-effects model for absolute agreement (ICC = 0.94, 95% CI 0.86–0.98). Bland–Altman analysis revealed a small positive bias of 2.1 mL, indicating slight overestimation by 3D-CE compared with CMR, with 95% limits of agreement from −19.0 to +18.7 mL. (*Figure 2–3*).

When categorizing RVol in the 21-patient subgroup into four severity levels (mild, moderate, moderate-severe, severe), CE-3D classified five patients as mild (23.8%), seven as moderate (33.3%), one as moderate–severe (4.8%), and eight as severe (38.1%). CMR classified four patients as mild (19.0%), seven as moderate (33.3%), two as moderate–severe (9.5%), and eight as severe (38.1%). The severity assignments showed strong concordance overall, with most disagreements limited to a single severity grade.

Among moderate and moderate–severe MR cases, 66.7% showed at least one marker of LV remodelling, defined as EDV/BSA > 100 mL/m², ECV > 29%, or T1 > 1050 ms. Nine patients exhibited multiple remodelling markers, with EDV/BSA > 100 mL/m² being the most frequently observed abnormality (*n* = 13). When comparing patients across MR grades, no statistically significant differences in remodelling markers were observed (all *P* > 0.05). This exploratory analysis was underpowered and should be interpreted as hypothesis-generating. Nevertheless, trends towards increasing LV size with greater MR severity and prolonged T1 values were observed, without reaching statistical significance (*P* = 0.0913).

## Discussion

Echocardiography is the most commonly used diagnostic modality for assessing MR. Based on this assessment, a critical clinical decision is made regarding whether to intervene or continue monitoring.^15^ MR quantification in patients with MVP can pose significant challenges due to factors such as mid-to-late systolic jet timing, multiple jets, and eccentric jets. Using 3D-CE to quantify MR in MVP patients may help overcome the limitations of other quantification methods.^16^ Traditional approaches, such as vena contracta and PISA-derived RVol, which rely on single-frame measurements during systole, may lead to overestimation. In this context, improved volumetric quantification using 3D-CE may help refine MR severity classification in patients with borderline or moderate MR, a subgroup in whom management decisions regarding timing of intervention vs. intensified surveillance are often challenging.

The primary source of error in calculating RVol using the CE arises from MV stroke volume-derived measurements. In contrast, Doppler flow assessment of the LVOT and the calculation of LVOT stroke volume are less prone to error and offer greater reproducibility compared to MA-derived measurements.^17,18^ Previous studies have demonstrated that the MA exhibits a complex anatomy in patients with MVP and MAD, differing significantly from that of normal subjects. MVP associated with MAD is characterized by distinct phenotypical features, including bileaflet prolapse, involvement of multiple scallops consistent with Barlow's disease, extensive leaflet redundancy, and paradoxical systolic enlargement. Furthermore, the MA is typically larger in these patients, with the inter-commissural diameter often longer than the anteroposterior diameter.^7,19^

**Figure 4:**
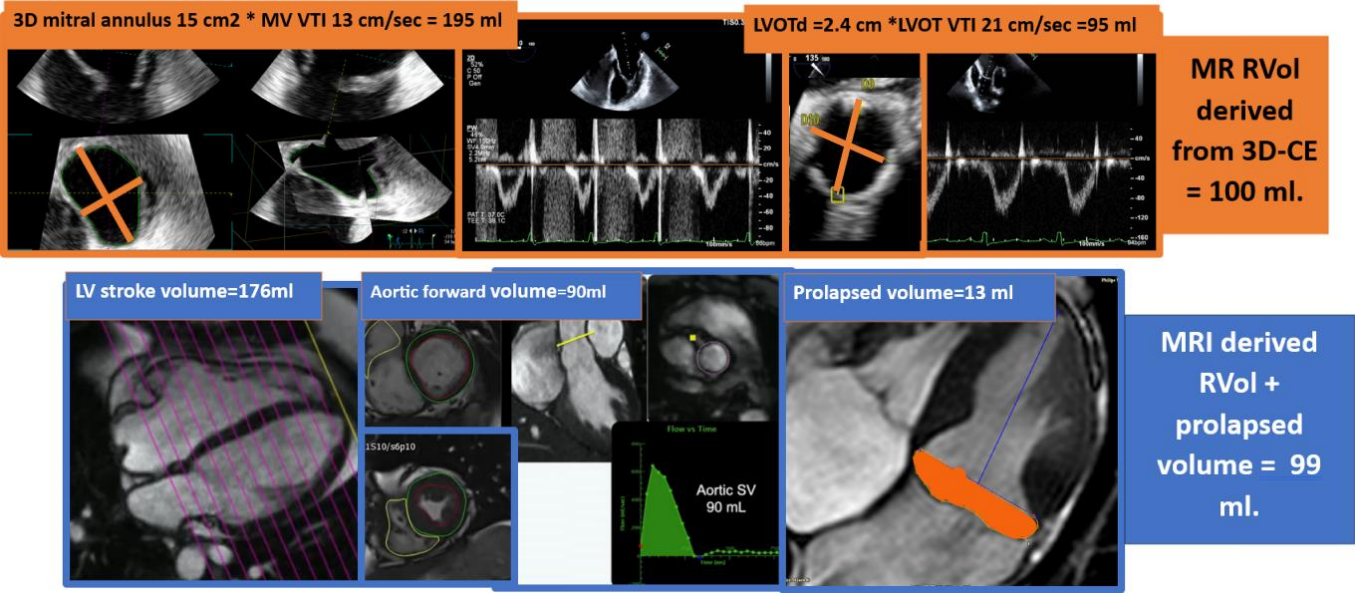
Schematic illustration comparing 3D mitral annular area measurement with diameter-derived annular area estimation.

Based on the discussion above, the assumption of a circular mitral annulus—particularly in cases of MVP with MAD is often inaccurate (*Figure 3, 4*). This limitation underscores the rationale for our approach: instead of relying on diameter-derived annular area calculations, we directly measured the area using 3D TEE datasets. This method provides a more precise and anatomically accurate representation of the MA geometry in these complex pathologies.

This was evident in our results, where the diameter-derived annular area overestimated the MA by 2.2 cm², leading to an average RVol overestimation of 19 mL compared with the directly measured 3D annular area. With the widespread adoption of 3D imaging, acquiring a 3D dataset of the mitral valve and aortic valve during TEE has become a routine in many centres, making the direct measurement of annular area a straightforward and readily accessible process. The advent of artificial intelligence software, such as Philips 3D Auto-CFQ, enables rapid and automated identification of the mitral and tricuspid annuli. This capability makes annular measurements more accessible and efficient, thereby facilitating the application of the 3D continuity equation for MR quantification.

While integrating 3D annular measurements improves geometric accuracy, it is crucial to recognize that several error sources remain to be acknowledged. Firstly, 3D datasets usually have lower temporal resolution than 2D imaging, which may result in decreased sensitivity, especially when using a 3D dataset from TTE rather than TEE or when the heart rate is irregular or high.^9^ Additionally, the MA is a highly dynamic, non-planar structure that undergoes substantial changes in shape throughout the cardiac cycle; as such, annular measurements taken at a single time point may not adequately capture this dynamic behaviour, even when derived from 3D datasets. Lastly, our approach does not eliminate the error associated with inaccurate Doppler alignment of the LVOT and MA, especially when suboptimal insonation angles are used.

Recently, one study examined the performance of the ASE algorithm of MR classification and the PISA technique against volumetric assessment by CMR. The ASE algorithm showed a low sensitivity of 56% and the PISA had a low specificity of 43%. On the contrary, echocardiography volumetric assessment showed better performance against CMR.^20^ When comparing 3D-CE to CMR volumetric RVol, there was good agreement, with perfect agreement in 80% of cases, increasing to 89.4% when accepting discordance of one grade of severity. Lopez-Mattei *et al*. compared TTE-derived CE using MA diameter to CMR RVol in patients with various MR mechanisms.^16^ Their results demonstrated a similar degree of agreement, despite significant differences between their population and ours. Our population consists of MVP patients with MAD, known for their complex MA anatomy and function. Uretsky *et al*. compared CMR with echocardiography for overall MR assessment and found only modest agreement, particularly in severe MR.^21^ This discrepancy may be attributed to differences in imaging modalities; Uretsky *et al*. used TEE in 38% of cases and primarily relied on the PISA method, which may contribute to variability in MR quantification. EROA derived from the PISA method, is commonly used as a marker to predict outcomes in patients with MR.^22^ In our study, one-third of patients had various MR jets, resulting in a modest correlation between PISA-derived RVol and CMR-derived RVol (*r* = 0.40).

Our observations are consistent with established literature demonstrating that echocardiographic LV volumes tend to be underestimated compared with CMR-derived measurements.^23^ CMR is considered the reference standard for LV volumetric assessment, and in patients with MVP, LV dilation predominantly reflects chronic volume overload related to MR. In addition, CMR provides unique insights into myocardial tissue composition, allowing detection of structural alterations such as myocardial fibrosis and extracellular matrix expansion.^24^ In this context, we explored the use of CMR-based tissue characterization parameters, including post-contrast T1 relaxation time and extracellular volume (ECV), alongside LV remodelling and dilation indices, as potential modifiers in patients with moderate MR. This analysis was exploratory and not powered to establish definitive associations or guide clinical decision-making. Nevertheless, our findings suggest trends towards greater LV dilation, prolonged T1 relaxation times, and higher ECV values in this subgroup, supporting the hypothesis that subclinical myocardial remodelling may already be present in selected patients with moderate MR. These observations should be considered hypothesis-generating and warrant confirmation in larger, prospective studies.

CMR and echocardiography are complementary imaging modalities for evaluating patients with MVP, rather than serving as alternatives to one another. Echocardiography, due to its widespread availability, cost-effectiveness, and real-time imaging capabilities, remains the first-line tool for initial screening and longitudinal follow-up of MVP patients with mitral MR. However, CMR provides superior precision in assessing LV volumes, quantifying MR severity, and evaluating RVol. Additionally, CMR offers advanced tissue characterization and the ability to detect early signs of LV remodelling, making it a valuable adjunct in comprehensive patient management.^25^

## Conclusion

In patients with MVP, using 3D-CE mitigates key geometric assumptions inherent to D-CE and, in a subgroup of 21 patients, improves agreement with CMR-based volumetric assessment. 3D-CE may be particularly valuable in patients with moderate or moderate-severe MR, in whom conventional echocardiographic parameters may be discordant. When interpreted within an integrative imaging framework, this approach has the potential to refine MR severity classification and support more confident guideline-directed clinical decision-making. Further prospective studies in larger cohorts are warranted to validate these findings and define the role of 3D-CE–based quantification in routine clinical practice.

### Limitations

This study has several limitations. Its retrospective design introduces potential selection bias and limits the ability to control for confounding variables. CMR was performed in most patients, but only 21 met the inclusion criterion of having CMR within 10 days of echocardiography and were included in the comparative analysis. As a result, estimates of agreement between 3D-CE–derived RVol and CMR volumetric assessment should be interpreted with caution, as the limited sample size may reduce the precision and generalizability of these findings. Differences in measurement techniques between 3D-CE-derived and CMR-derived RVol may introduce measurement variability, and, as a single-centre study, the findings may not be fully generalizable to other populations or clinical settings.

## Lead Author Biography


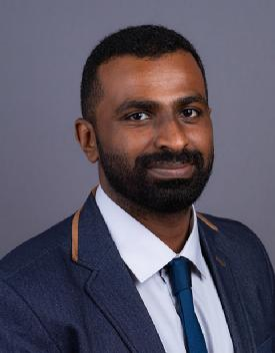


Abdalla Eltayeb Abdalla Abdelkader, MD, is an Assistant Professor in the Division of Cardiovascular Medicine, Department of Medicine, College of Medicine at the University of Florida. His clinical and research interests focus on echocardiography and multimodality cardiac imaging, with particular expertise in mitral valve prolapse and valvular heart disease. He is actively engaged in advancing quantitative imaging approaches to improve the assessment and management of complex valvular disorders.

## Data Availability

The data underlying this article were generated from clinical imaging studies and contain sensitive patient information. Due to ethical and privacy considerations, the data are not publicly available. De-identified data may be made available from the corresponding author upon reasonable request and subject to institutional approval.
